# HMGB1-TLR4-IL23-IL17A axis promotes paraquat-induced acute lung injury by mediating neutrophil infiltration in mice

**DOI:** 10.1038/s41598-017-00721-8

**Published:** 2017-04-04

**Authors:** Bailing Yan, Feng Chen, Lijun Xu, Jihong Xing, Xuefu Wang

**Affiliations:** 1 Emergency Department, the First Hospital of Jilin University, Changchun, 130021 China; 2 Dermatology Department, China-Japan Union Hospital of Jilin University, Changchun, 130033 China; 3 Department of Respiratory Medicine, the First Hospital, Jilin University, Changchun, 130021 China; 4 School of Pharmacy, Anhui Medical University, Hefei, Anhui 230032 China; 5 Institute of Immunology, School of Life Sciences, University of Science and Technology of China, Hefei, Anhui 230027 China

## Abstract

Paraquat is a poisoning herbicide that primarily targets lung, leading to severe acute lung injury characterized by extensive neutrophil infiltration. However, the mechanisms underlying the neutrophil infiltration is not clear. In this study, we demonstrated the significance of the signaling cascade from high-mobility group box 1 (HMGB1), to Toll-like receptor 4 (TLR4), interleukin-23 (IL-23), and lastly to IL-17A during the paraquat-induced neutrophil infiltration and the subsequent lung injury in mice. Paraquat challenge significantly elevated serum levels of IL-17A and IL-23, the percentage of IL-17A-producing γδT cells in the lung, and the level of HMGB1 in bronchoalveolar lavage fluid. Reducing IL-17A production using an anti-γδT antibody, targeting IL-23 with the neutralizing antibody against IL-23p19, and blocking HMGB1 signaling by using glycyrrhizin or TLR4^−/−^ mice all dramatically inhibited the infiltration of neutrophils and attenuated lung injury. These novel findings not only reveal the critical role of HMGB1-TLR4-IL-23-IL-17A axis in the pathogenesis of paraquat-induced acute lung injury, but also provide promising therapeutic targets for treating paraquat poisoning.

## Introduction

Paraquat (1,1′-dimethyl-4,4′-bipyridinium dichloride, PQ) is a commonly used herbicide and a leading cause of fatal poisoning worldwide, particularly in developing countries^1–6^. Without known antidote available, PQ poisoning has become a severe public health problem in some countries^1–4^. Although multiple organs including liver, kidney, heart and central nervous system are frequently inflicted, lung is the primary target of PQ. In the lung tissues, PQ is actively taken up against a concentration gradient and accumulates at particularly high levels in Clara cells, as well as in alveolar type I and II epithelial cells^7^, leading to acute lung injury(ALI) and the subsequent respiratory failure that becomes most common cause of death from PQ^8, 9^. Infiltration by a significant number of neutrophils accompanies the early pathological changes of PQ poisoning, including pulmonary edema, hemorrhage, and/or interstitial inflammation. However, the mechanism regulating the infiltration of these neutrophils is largely unknown.

Interleukin 17 A (IL-17A) is a pro-inflammatory cytokine critically regulating the host defense against multiple pathogens^10^. Controlling the recruitment of neutrophils and other immune cells to the infection site is a major mechanism underlying IL-17A activities^11^. Although T helper (Th)17 cells are considered as the major cells for producing of IL-17A, other innate immune cell populations including NK cells, γδ T cells, and even neutrophils are also known to secrete IL-17A^12^. Recent studies showed that γδT cells play an important role in aseptic inflammation and autoimmune diseases in an IL17A-dependent manner; particularly, IL-17A-producing γδ T cells contribute to the acute live injury induced by Acetaminophen^11^. The production of IL-17A is essentially controlled by IL-23, a heterodimeric cytokine comprising a p19 unit and a p40 subunit through multiple mechanisms. First, IL-23 stimulates the differentiation of Th17 cells from naïve CD4^+^ T cells^13^. Second, IL-23, together with anti-CD3, triggers IL-17A production from NKT cells^14^. Third, IL-23 works with IL-1 to release IL-17A from γδ T cells^15^. Functionally, the IL-23/IL-17A axis plays an important role in the development of inflammation and autoimmune diseases, and is becoming a potential therapeutic target for the treatment of these conditions.

The upstream molecular control of the IL-23-IL-17A axis is not completely understood. Recent studies suggest that the high-mobility group box 1 (HMGB1), a chromatin-binding protein that can be secreted by necrotic cells or inflammatory cells, may act through multiple receptors, including toll-like receptor (TLR)2, TLR4, TLR9, or the receptor for advanced glycation end products (RAGE), to stimulate IL23 production and activate the IL-23-IL-17A axis^11, 16–18^.

In this study, we hypothesized that the neutrophil infiltration as witnessed during ALI development of PQ poisoning is regulated by IL-17A, which is further controlled through the HMGB1-TLR4-IL-23-IL-17A axis. Therefore, targeting this axis will present a potential therapeutic strategy for treating PQ poisoning or other ALI-involved diseases. To test this hypothesis, we established a mouse model of PQ poisoning and applied loss-of-function approaches to evaluate the significance of targeting the HMGB1-TLR4-IL-23-IL-17A axis in neutrophil recruitment and ALI development in response to PQ challenge.

## Materials and Methods

### Mice

All experimental protocols were approved by the Institutional Ethics Committee for Animal Use in Research of University of Science and Technology of China (USTC; Hefei, China) and the methods were carried out in accordance with Animal Care Guidelines of USTC. C57BL/6 male mice between 6 to 8 weeks were purchased from the Shanghai SLAC Laboratory Animal center (Shanghai, China). TLR4 knockout (*TLR4* ^−/−^) mice on the C57BL/6 background were kindly provided by Dr. Shaobo Su (School of Medicine, Tongji University, Shanghai, China). The mice were housed at room temperature of (22 ± 1) °C on a 12/12-hr light/dark cycle in the specific pathogen-free facility of the School of Life Sciences (USTC) with food and water provided *ad libitum*.

### Animal model of PQ poisoning

PQ (Sigma-Aldrich, St. Louis, MO, USA) was dissolved to a concentration of 10 mg/mL in phosphate-buffered saline (PBS). To induce PQ poisoning, mice were gavaged with PQ at 40 mg/kg (body weight). As the vehicle control, equivalent volume of PBS was administered intragastrically. To block endogenous IL-17A or IL-23 *in vivo*, 0.2 mg blocking antibody for mouse IL-17A blocking antibody (BioLegend, San Diego, CA, USA) or for mouse IL-23p19 (eBioscience, San Diego, CA, USA) was injected intravenously (*i.v*.) one hour before the mice was gavaged. Isotype-matched IgG was used as control. To deplete endogenous γδ T cells, 0.5 mg of an anti-γδTCR mAb (ATCC, Manassas, VA) was *i.v*. injected at 48 hours before PQ administration. To inhibit endogenous HMGB1, glycyrrhizin (TCI, Shanghai, China) was administered *i.v*. into mice at 5 mg per mouse every day for 3 consecutive days before PQ administration. At 72 hours after PQ or PBS gavage, all mice were euthanized. The lungs were removed and divided into different aliquots for further analysis (see below).

### Measurement of total protein level in bronchoalveolar lavage fluid (BALF)

To evaluate vascular permeability in the airways, BALF was collected as previously described^19^ with modifications. Briefly, BALF was obtained by rinsing lungs with 0.8 ml PBS followed by centrifugation at 1500 rpm for 5 min. The supernatants were collected, with the total protein level (mg/mL BALF) measured by BCA method.

### Measurement of cytokines

The levels of cytokines TNF-a, IL-1β, IL-6, CXCL1 and MMP-9 in the BALF were measured by using mouse ELISA kits from Abcam (Cambridge, UK) and multi sciences (shanghai, China) according to the manufacturer’s instructions.

### Determination of lung wet-to-dry weight (W/D) ratio

To assess the extent of lung edema/water accumulation, the lung weight was immediately measured upon isolation from the mice and repeated after being dried in an oven at 60 °C for 24 h.

### Determination of pulmonary myeloperoxidase activity (MPO)

To quantify the extent of neutrophil infiltration into the lung, the MPO activity was measured on frozen lung tissue using the MPO activity test kit (Nanjing Jiancheng Bioengineering Institute, Nanjing, China), according to the manufacturer’s instructions.

### Histological analysis

Lung specimens were fixed in 4% paraformaldehyde, dehydrated through alcohol series of increasing concentrations, embedded into paraffin, and prepared into sections of 6-µm thickness. After staining with hematoxylin and eosin (HE), the slides were evaluated under a light microscope (Olympus BX51, Tokyo, Japan,) by board-certified pathologist who was blind to genotypes of or treatments the mice received. The severity of lung damage was scored according to the following histological parameters: alveolar wall thickness, the amount of cellular infiltration and hemorrhage. The histological parameters were graded on a scale of 0–3 (0 = none, 1 = mild, 2 = moderate, 3 = severe).

### Quantitative Reverse-Transcription Polymerase Chain Reaction (RT-PCR)

Total RNA was extracted from frozen lung tissues using Trizol (Invitrogen, Carlsbad, CA, USA) following the manufacturer’s instructions and reverse transcribed into cDNA. Real-time PCR was performed in a Corbett Rotor-Gene 3000 real-time PCR system (Corbett Research, Sydney, Australia) using the specific primers for IL-17A, IL-23p19, and β-actin (internal control)^11^ and SYBR Premix Ex Taq kit (Takara, Shiga, Japan) according to the manufacturer’s instructions. The PCR reaction conditions were 45 cycles of 95 °C for 10 seconds followed by 60 °C for 30 seconds. The quantification of a target gene expression relative to β-actin was achieved by using the 2^(−ΔΔCt)^ method^20^.

### Measurement of serum IL17A, IL23, and HMGB1

The serum levels of cytokines were measured using the enzyme-linked immunosorbent assay (ELISA) kits for IL-17A (Dakewe Biotech, Shenzhen, China), IL-23 (Biolegend), and HMGB1 (Yanhui Biotech, Shanghai, China), respectively, according to the manufacturer’s instructions.

### Isolation of pulmonary leukocytes

Pulmonary leukocytes were isolated as previously described^19^ with modifications. Briefly, lungs were excised from the mice, minced into small pieces, and digested for 60 min at 37° in RPMI-1640 medium containing 0.1% collagenase I (Sigma) and 5% fetal calf serum. After filtering out the non-digested tissue chunks through gauze (200G), the red blood cells (RBCs) were lysed using the RBC lysis buffer (Biolegend). The total leukocyte and neutrophils number was counted on the Sysmex XE-5000 (Sysmex, Kobe, Japan)^21^.

### Flow cytometric analysis

To analyze the IL-17A-producing γδ T cells in lung tissue, the cells were blocked by rat serum, followed by activation at 37 °C for 4 h with 50 ng/mL Phorbol 12-myristate 13-acetate (PMA),1 μg/mL ionomycin, and 10 μg/mL monensin (Sigma). After being stained with fluorophore-labeled antibodies for extracellular markers, the cells were fixed, permeabilized, and stained with fluorophore-labeled antibodies against intracellular cytokines. Isotype-matched IgG antibodies were used as controls. Samples were then passed through an LSRII flow cytometer (BD Biosciences, San Jose, CA, USA) and analyzed by FlowJo software (FlowJo 7.6.1, USA). The following fluorophore-conjugated anti-mouse monoclonal antibodies (mAbs) were used in this study: FITC-CD45, PE-CD3,PE-Cy7-CD3, Percy-Cy5.5-RORγt, Percp-Cy5.5-IL-17A, and APC-γδTCR (all from BD Biosciences).

### Statistical Analysis

Data were presented as mean ± stand deviation of the mean (SD). The significance of differences was determined using a two-tailed unpaired *t* test and the significance levels were marked *P < 0.05; **P < 0.01; ***P < 0.005.

## Results

### PQ gavage induces ALI in mice

**Figure 1:**
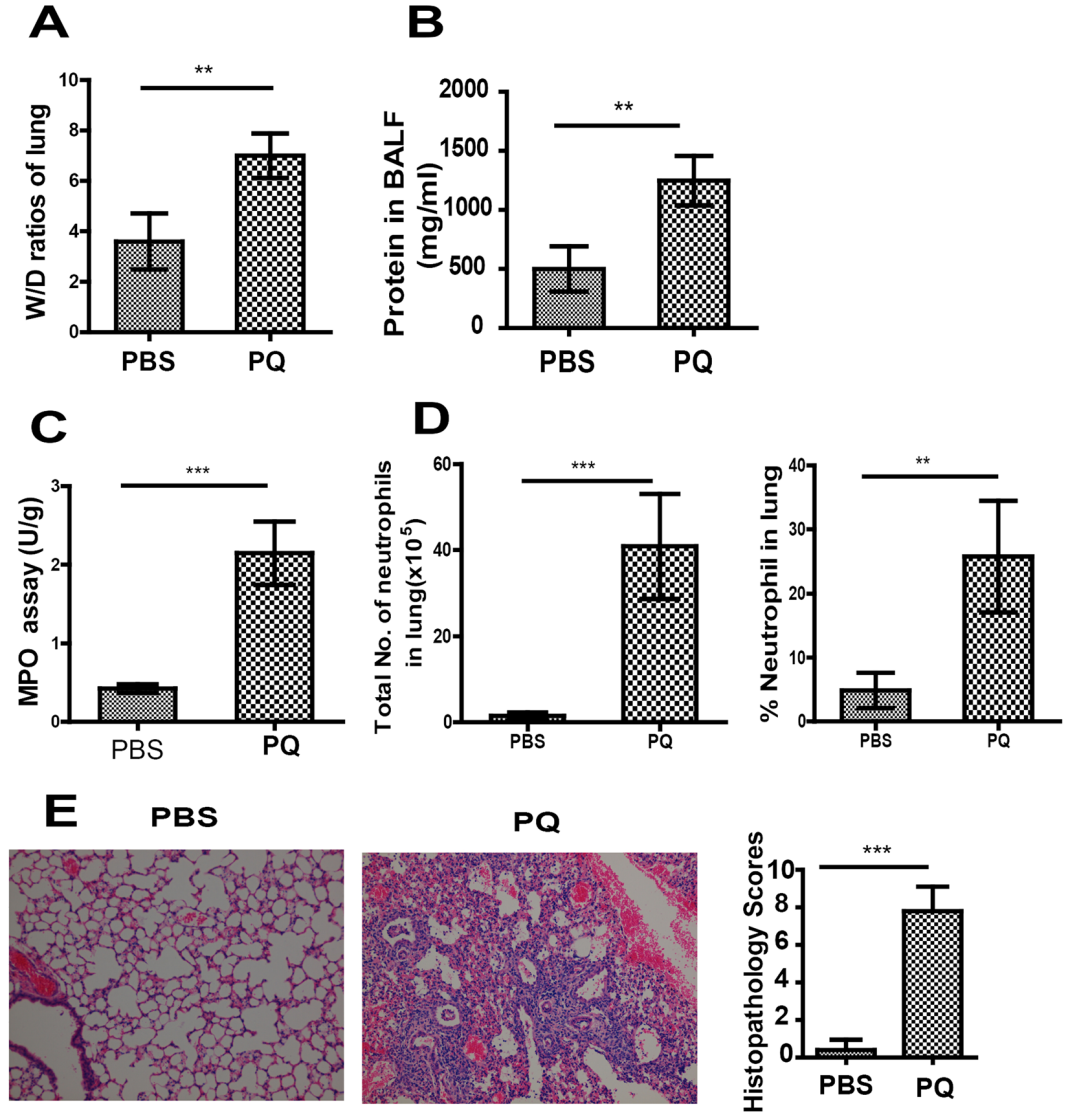
Paraquat (PQ) ingestion induces acute lung injury (ALI) and neutrophil infiltration into the lung. Mice were gavaged with PQ at 40 mg/kg (body weight; n = 15) or equal volumes of PBS as the vehicle control (n = 15). At 72 h after PQ challenge, the mice were sacrificed, and the lung tissue or bronchoalveolar lavage fluid (BALF) was collected for analyzing the following parameters indicating lung injury. (**A–D**) The wet to dry (W/D) ratio of the lung (**A**), the total protein concentration (mg/ml) in BALF (**B**), the MPO activity with the lung tissues (**C**), the total number and percentage of lung-infiltrating neutrophils (**D**) were significantly elevated in PQ-challenged mice, compared to PBS-challenged control mice. (**E**) Hematoxylin and Eosin (H & E) staining of the lung tissues (200×) presented a higher histopathology score in PQ-challenged mice. All data are presented as mean ± SD for all mice in each group and compared between the PQ- and PBS-challenged mice. **P < 0.01, ***P < 0.005, compared to PBS-challenged mice.

To explore the molecular mechanisms underlying PQ-induced ALI, we intragastrically administered indicated dose of PQ into the mice and assessed multiple parameters related to ALI at 72 h after PQ gavage. PQ at the dose of 40 mg/kg could induce acute lung injury (Fig. S1A). As shown in Fig. 1, all parameters examined, including the W/D ratio (a measure of lung edema, Fig. 1A), total protein level in BALF (an indicator for alveolar epithelial integrity, Fig. 1B), and MPO activity (a gauge of neutrophil activity, Fig. 1C) were significantly elevated in PQ-challenged mice, compared to PBS-challenged control mice (all P < 0.01), suggesting the presence of ALI in PQ-challenged mice. On the cellular level, we detected significant increase of total neutrophils in the PQ-challenged lung tissue, compared to the corresponding levels in PBS-challenged mice (Fig. 1D; P < 0.001). Consistently, we observed more severe lung damage in PQ- but not PBS-challenged mice (Fig. 1E; P < 0.001). On the molecular level, the administration of PQ increased the levels of TNF-a, IL-1β, IL-6, CXCL1 and MMP-9 in the BALF when compared with PBS (Fig. S1B,C). Taken together, these results demonstrate PQ challenge can induce severe ALI and neutrophil infiltration in the lung.

### IL17A critically controls PQ-induced ALI

**Figure 2:**
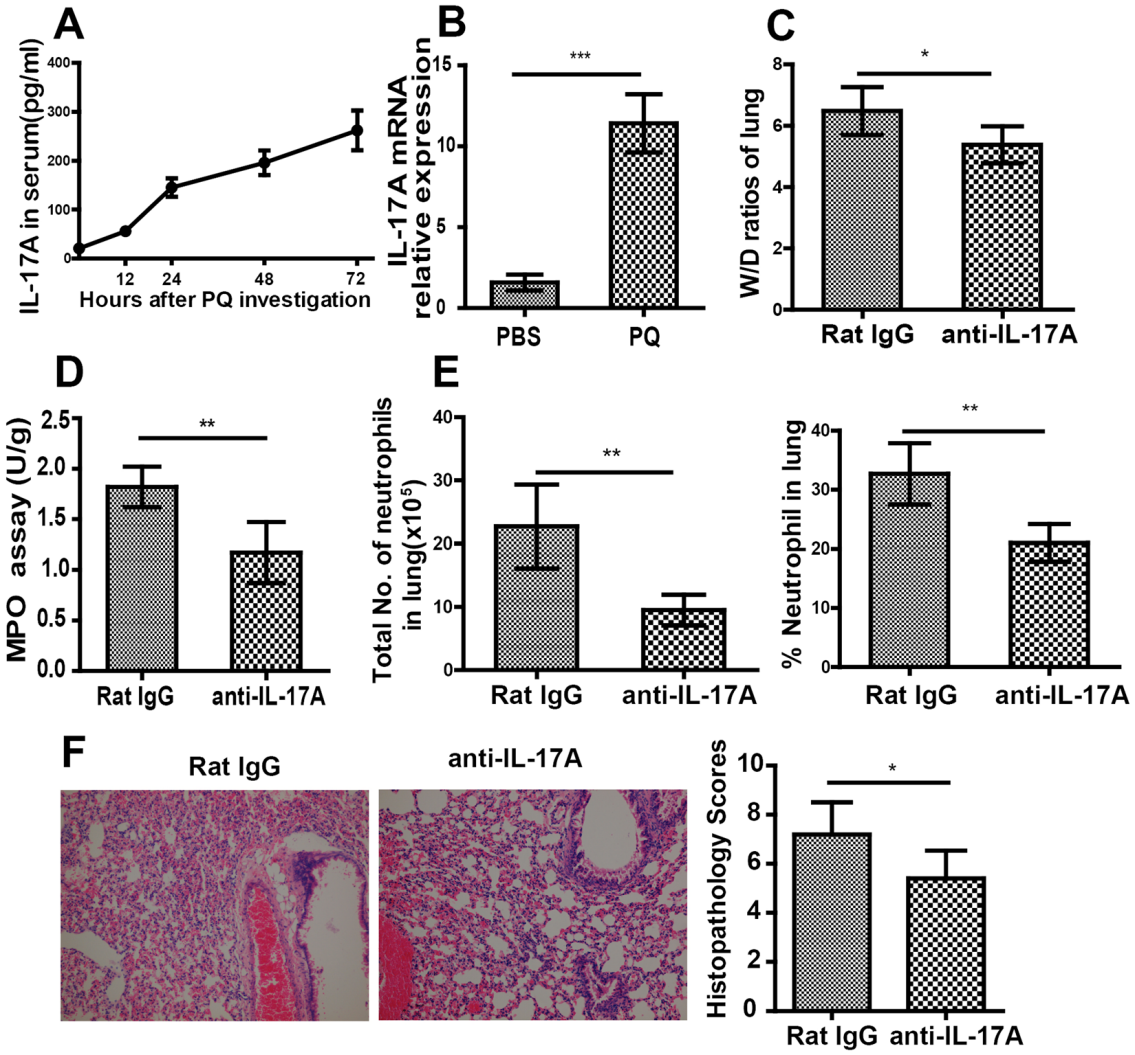
IL-17A is essential for neutrophil infiltration and ALI development in response to PQ. Mice were intravenously injected with either neutralizing antibody for IL17A (anti-IL17A; n = 6) or isotype-matched control IgG (rat IgG; n = 6) at one hour before they received PQ gavage. At 72 h after PQ challenge, the mice were sacrificed and lung tissues were collected. (**A**) Serum IL-17A level increased in a time-dependent manner following PQ challenge. At indicated time points after PQ gavage, the serum IL-17A levels were determined by ELISA. (**B**) IL-17A expression in the lung was up-regulated in response to PQ poisoning. The expression of IL-17A in the lung at 72 h after PQ challenge was determined by RT-PCR. (**C–F**) The W/D ratio (**C**), MPO activity (**D**), number and percentage of lung-infiltrating neutrophils (**E**) were significantly reduced in anti-IL-17A-treated mice, when compared to those treated with Rat IgG. (**F**) H&E staining (200×) revealed less severe lung damage in anti-IL-17A-treated mice than in Rat IgG-treated mice. All data are presented as mean ± SD for all mice in each group and compared between PQ- and PBS-challenged mice (A and B) or between the anti-IL17A- and rat IgG-injected mice (C to G). *P < 0.05, **P < 0.01, and ***P < 0.005.

To examine whether IL-17A mediates neutrophil infiltration in PQ-induced ALI, we first monitored the serum levels of IL-17A at different time points after PQ gavage. We found that the serum IL-17A level increased in a time-dependent manner following PQ challenge (Fig. 2A). In addition, the IL-17A expression was significantly up-regulated in the lung tissue at 72 h after PQ challenge, when compared to the level in the lungs from PBS-challenged mice (P < 0.001; Fig. 2B), suggesting the injured lung tissue could be a source for serum IL17A following PQ challenging. Most importantly, pre-treatment of mice with IL17A blocking antibody dramatically alleviated pathological changes associated with PQ-induced ALI, as demonstrated by significant reductions in the W/D ratio (Fig. 2C, P < 0.05), MPO activity (Fig. 2D and Fig. S2A, P < 0.01), and the percentage as well as number of neutrophils infiltrating into the lung tissues (Fig. 2E, P < 0.01) (when compared to mice pre-treated with isotype-matched control rat IgG). In addition, the administration of anti-IL-17A significantly decreased the levels of TNF-a, IL-1β, IL-6, CXCL1 and MMP-9 in the BALF when compared to the corresponding levels from Rat IgG-treated mice (Fig. S2B,C). The attenuations in ALI pathologies in response to IL17A-blocking antibody were also reflected in lung histology (Fig. 2F, P < 0.05). Taken together, our data show that IL-17A is essential for neutrophil infiltration and PQ-induced ALI.

### The pulmonary γδ T cells are a significant source for IL-17A

**Figure 3:**
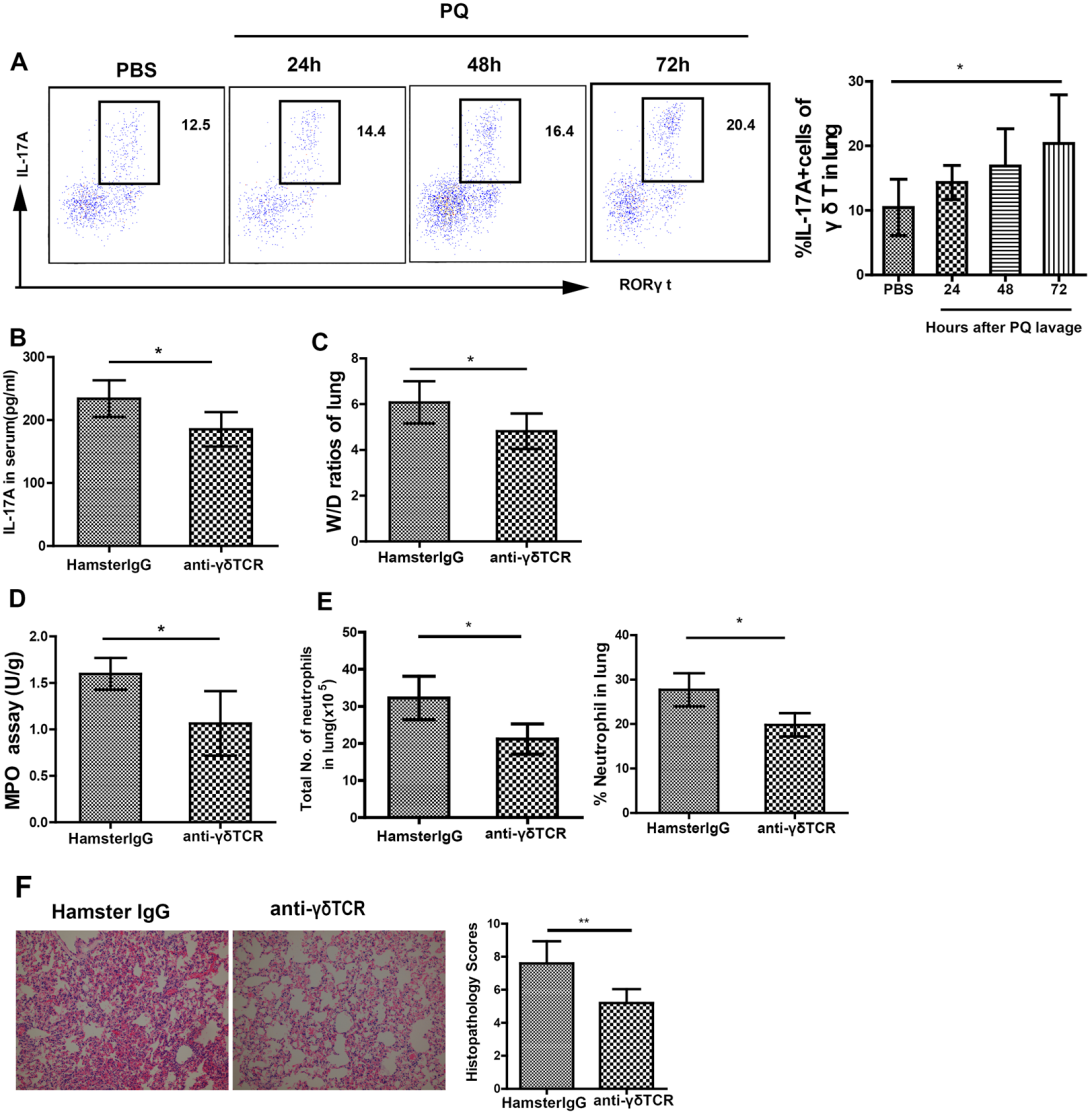
γδT cells are a significant source for IL-17A production during PQ-induced ALI. (**A**) PQ poisoning significantly increased the percentage of IL17A^+^ γδ T cells in the lung. Mice were intravenously injected with either anti-γδTCR antibody for depleting endogenous γδ T cells (anti-γδTCR; n = 6) or isotype-matched control IgG (hamster IgG; n = 6) two days before they received PQ gavage. At 72 h after PQ challenge, the mice were sacrificed and serum or lung tissues were collected. (**B–F**) Level of IL-17 in the serum (**B**), the W/D ratio (**C**), the MPO activity (**D**), the number and percentage of lung-infiltrating neutrophils (**E**), and the histopathology score (200×; (**F**) were significantly lower in anti-γδTCR group than in the control hamster IgG group. All data are presented as mean ± SD for all mice in each group and compared between the PQ- and PBS-challenged mice (**A**) or between the anti-γδTCR- and hamster IgG-injected mice (B to F). *P < 0.05 and **P < 0.01.

To identify the cell sources for IL-17A, we observe the infiltration kinetics of γδ T cells and Th17 cells in the lung with PQ injury. The result show that the percentage of IL-17A^+^ γδ T cells increased from approximately 10% to more than 20% at 72 h after PQ challenge (Fig. 3A) while the percentage of Th17 cells did not significantly change (Fig. S3C). After the mice were pre-treated with anti-γδTCR antibody to deplete *in vivo* γδ T cells, the level of serum IL-17A in response to PQ challenge was significantly reduced (P < 0.05, when compared to mice pre-treated with isotype-matched Hamster IgG; Fig. 3B). In addition to inhibition of serum IL-17A level, depletion of γδ T cells also decreases the W/D ratio, MPO activity, the number of infiltrating neutrophils in the lung tissues (P < 0.05; Fig. 3C,D,E), as well as attenuated histological injury (P < 0.01; Fig. 3F). Taken together, these data suggest that γδ T cells are significant producers for IL-17A in PQ-induced ALI.

### IL-23 is required for the production of IL-17A from γδ T cells

**Figure 4:**
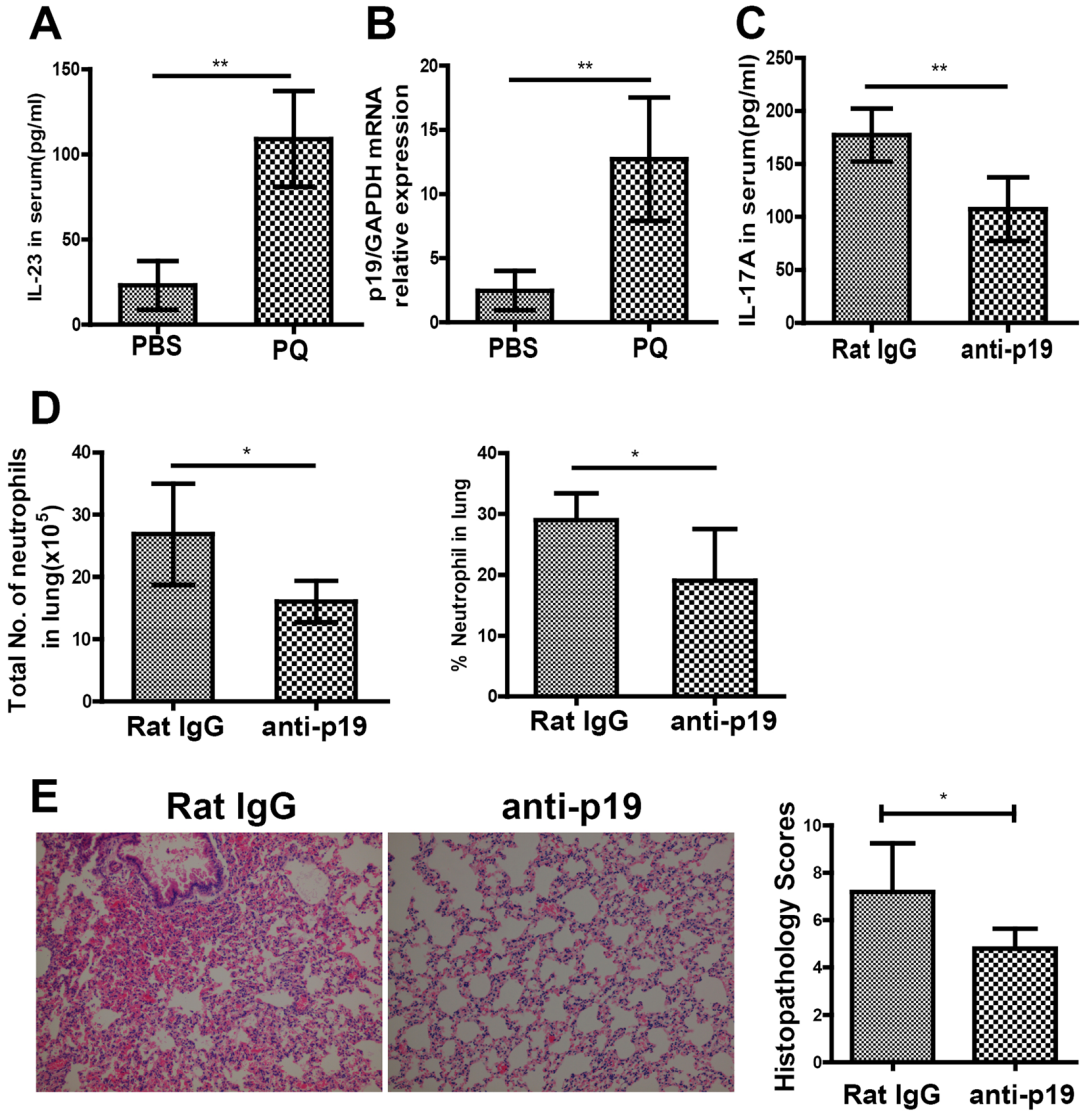
IL-23 is essential for the production of IL-17A and neutrophil infiltration into the lung in response to PQ challenge. (**A**,**B**) At 72 h after PQ or PBS gavage, the serum IL-23 level as measured by ELISA (**A**), and the steady-state mRNA level of IL-23 in the lung tissue detected by RT-PCR (**B**) were significantly higher in PQ group than in PBS group. Mice were intravenously injected with either neutralizing antibody for IL-2319p (anti-19p; n = 6) or isotype-matched control IgG (rat IgG; n = 6) at one hour before they received PQ gavage. At 72 h after PQ challenge, the mice were sacrificed, and serum and lung tissues were collected. (**C**,**D**) Pre-treatment with anti-19p significantly reduced serum IL-17A level (**C**), the No. and percentage of lung-infiltrating neutrophils (**D**), and significantly decreased histopathology score of the lung tissue (**E**) than in mice pre-treated with control rat IgG. All data were presented as mean ± SD for all mice in each group and compared between the PQ- and PBS-challenged mice (A and B) or between the anti-19p- and rat IgG-injected mice (C, D, and E). *P < 0.05 and **P < 0.01.

Previous studies showed that in multiple disease models, the production of IL-17A from γδ T cells is dependent on IL-23^11^. To examine whether IL-23 was required for the production of IL-17A in PQ-induced ALI, we first measured the serum levels as well as the expression of IL-23 in the lung tissue at 72 h after PQ challenge. Both analyses showed dramatic increase of IL-23, when compared to PBS-challenged mice (P < 0.01; Figs. 4A and B). When blocking the endogenous IL-23 with the neutralizing antibody targeting the p19 subunit, both the serum IL-17A level (P < 0.01, Fig. 4C) and the number of neutrophils (P < 0.05, Fig. 4D) infiltrating into the lung tissue, as well as attenuated histological injury (P < 0.05; Fig. 4E) were significantly reduced, as compared to mice pre-treated with isotype-matched rat IgG. In consistency, anti-p19 decreased the levels of TNF-a, IL-1β, IL-6, CXCL1 and MMP-9 in the BALF (Fig. S4). Taken together, our data show that IL-23 is required for γδ T cells to generate IL-17A in PQ-induced ALI.

### HMGB1 signals through TLR4 to promote the release of IL-23 in response to PQ challenge

**Figure 5:**
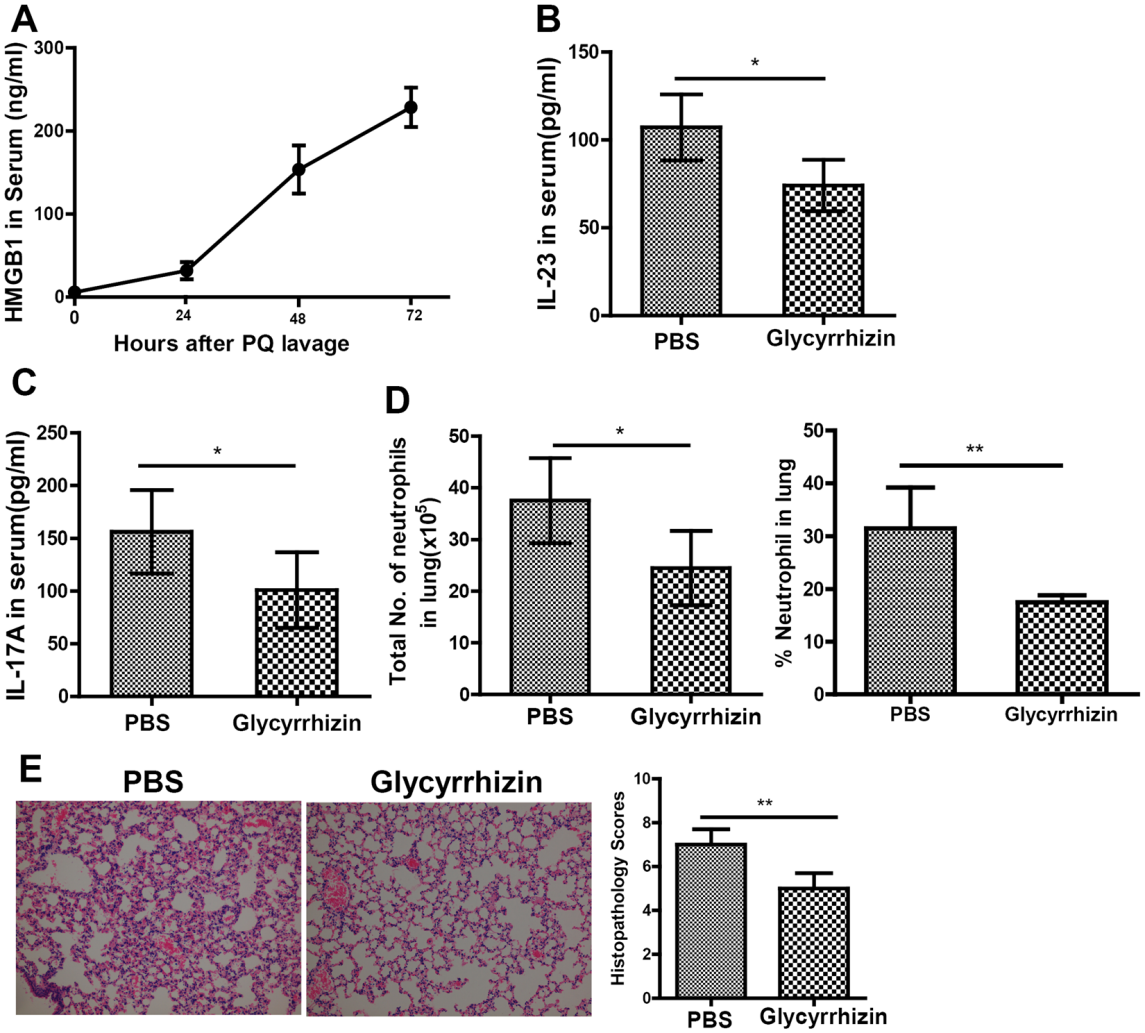
HMGB1 critically controls the production of IL-23 and IL-17A, as well as neutrophil infiltration into the lung in response to PQ challenge. (**A**) At indicated time points after PQ or PBS gavage, the amount of HMGB1 in Serum increased in a time-dependent manner. Mice were administered *i.v*. with glycyrrhizin or equal volume of PBS for 3 days after PQ gavage. At 72 h after PQ challenge, the mice were sacrificed, and serum and lung tissues were collected. (**B–D**) Glycyrrhizin significantly reduced serum IL23 level (**B**), serum IL17A level (**C**), the total number and percentage of neutrophils in the lung (**D**), and lung injury as represented by histopathology score (200×) (**E**). All data were presented as mean ± SD for all mice in each group and compared between the glycyrrhizin- and PBS-treated mice. *P < 0.05, **P < 0.01.

**Figure 6:**
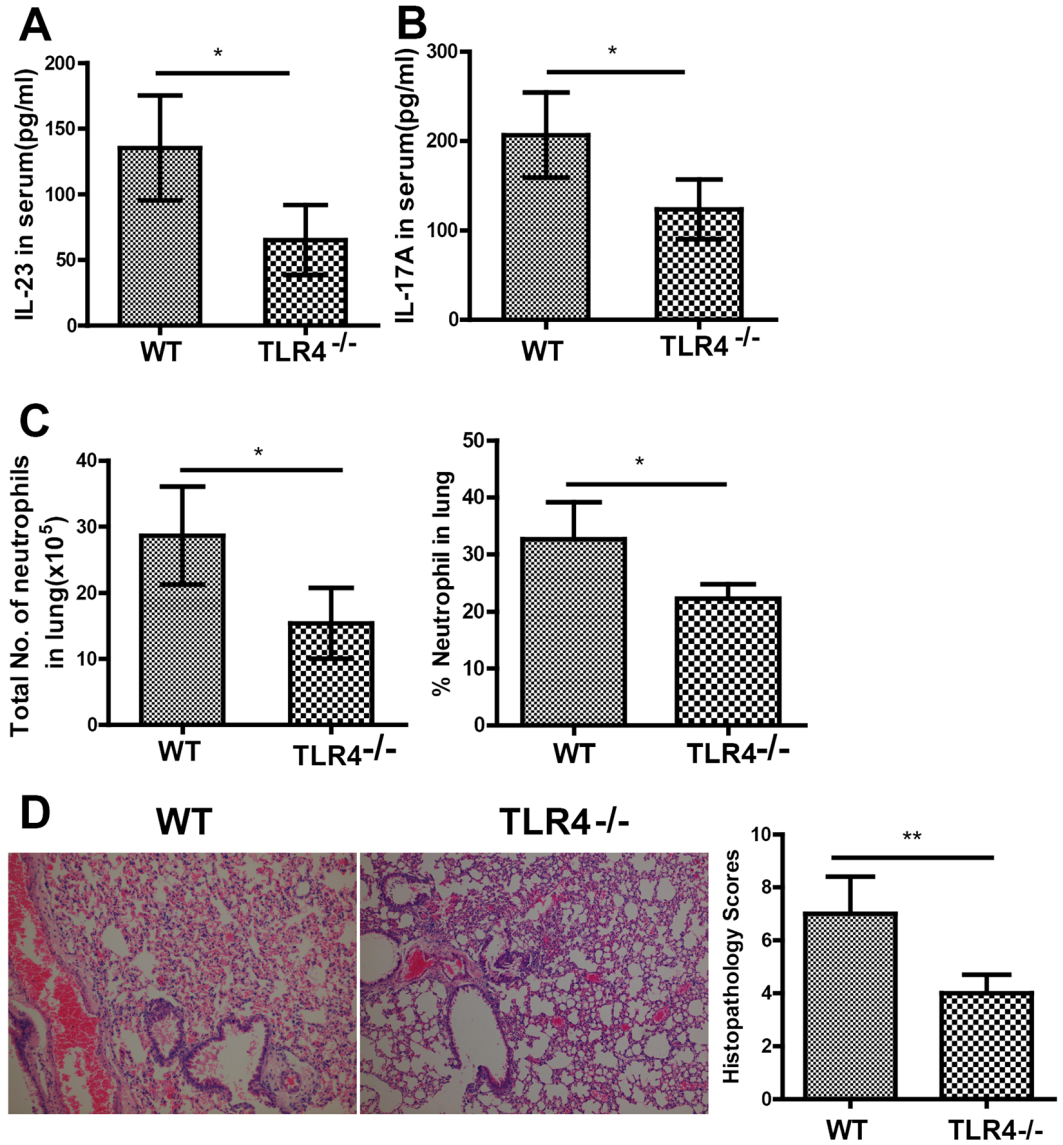
TLR4 mediates the activation of HMGB1-IL-23-IL-17A axis following PQ challenge. PQ gavage was performed on TLR4^−/−^ or wild-type (WT) C57BL/6 mice. At 72 h after PQ challenge, TLR4^−/−^ group presented significantly lower levels of the serum IL-23 (**A**), the serum IL-17A (**B**), the number and percentage of lung-infiltrating neutrophils (**C**), and the lung injury (200×) (**D**). All data were presented as mean ± SD for all mice in each group and compared between the TLR4^−/−^ and WT mice. *P < 0.05.

HMGB1 is a damage-associated molecular pattern (DAMP) molecule that is released by damaged tissue and capable of initiating innate as well as adaptive immune responses^22^. To assess the potential involvement of HMGB1 in IL-23-IL-17A-mediated neutrophil infiltration in response to PQ poisoning, we first monitored the release of HMGB1 in serum, BALF and gut tissues at indicated time points after PQ challenge. Similar to the pattern of serum IL-17A following PQ challenge (Fig. 2A), the amount of HMGB1 increased in a time-dependent manner in PQ-challenged mice (Fig. 5A), correlated with the level in BALF (Fig. S5A). However, the levels of HMGB1 in the gastric and intestinal tissues did not significantly change (Fig. S5B). When the mice were pre-treated with HMGB1 inhibitor, glycyrrhizin, the serum levels of IL-23, IL-17A, as well as the number of infiltrating neutrophils in the lung tissue were all significantly inhibited (P < 0.05; Fig. 5B to D), which correlated with improved changes on histology (P < 0.01; Fig. 5E). Similarly, the administration of Glycyrrhizin was associated with the levels of TNF-a, IL-1β, IL-6, CXCL1 and MMP-9 in the BALF (Fig. S5C,D). It is known that HMGB1 may signal through TLR2, TLR4, TLR9, or RAGE, to stimulate IL23 production and activate the IL-23-IL-17A axis^18, 23–25^. To assess the significance of TLR4 in the activation of IL-23-IL-17A axis in response to PQ challenge, we administered PQ to either TLR4^−/−^ or wild-type (WT; as controls) C57BL6 mice. We found that the serum levels of IL-23 (Fig. 6A) and IL-17A (Fig. 6B) were significantly lower in TLR4^−/−^ mice than in WT mice (P < 0.05), which was associated with dramatically lower number of neutrophils (Fig. 6C) in the lung tissue (P < 0.05) and improved changes on histology (P < 0.01; Fig. 6D). Collectively, these data support that HMGB1-TLR4 signaling mediated the production of IL-23 in PQ-induced ALI.

## Discussion

ALI is a major consequence of PQ poisoning, leading to significant morbidity and frequently to death. The infiltration of neutrophils into the lung tissue, through the productions of numerous cytotoxic substances, including granular enzymes, reactive oxygen species, and various pro-inflammatory cytokines, critically controls the development and severity of ALI^26–28^. Therefore, blocking neutrophil infiltration provides a promising therapy for PQ-induced ALI. In this study, we identified, for the first time, that the HMGB1-TLR4-I-L23-IL-17A axis plays a critical role in regulating neutrophil infiltration and ALI development following PQ poisoning. Targeting the components in this axis could potently inhibit neutrophil infiltration and alleviate ALI symptoms.

PQ is actively taken up against a concentration gradient into lung tissue leading to ALI, and eventually to cell death^29–31^. In rat models of PQ poisoning, it has been shown that multiple inflammatory factors, including TNFα, IL-1β, IL-6, and HMGB1 were elevated in the lung tissue or serum^29, 30^, suggesting the development of an acute immune response following PQ-induced cell death. Consistent with these findings, we showed here that the DAMP molecule, HMBG1 is increasingly released in a time-dependent manner into the lung tissue in response to PQ poisoning to mice, which is associated with the increased serum levels of pro-inflammatory cytokines, IL-23 and IL-17A, and the enhanced neutrophil infiltration into the lung tissue. More importantly, we showed that blocking HMGB1 with glycyrrhizin significantly attenuates the immune responses and lung injury induced by PQ, suggesting that DAMP-mediated immune response constitute a second wave of damages to the lung tissue, as have been noticed for many drug toxicity^31^. However, Lei *et al*. recently showed that in myocardial dysfunction induced by PQ, although IL-1β and phosphorylated IκB levels were up-regulated, other pro-inflammatory markers including TNFα, HMGB1, MyD88, and p53 did not respond to PQ challenge^32^, suggesting that the inflammatory mediators for PQ poisoning vary by the target organs, and thus differential therapies may be applied depending on which organs are inflicted. Since lung is the major target for PQ poisoning, it is critical to understand the pathogenic mechanisms underlying PQ-induced cytotoxicity as well as the subsequent immune damages in lungs.

HMGB1 is known to signal through multiple TLR members and RAGE, depending on the disease paradigm^33^. Specifically, TLR4 is considered a pattern-recognition receptor in ALI and is extensively demonstrated to mediate HMGB1-induced lung injury^34, 35^. Liu *et al*. showed that PQ up-regulates TLR4 expression, which is associated with the elevation of pro-inflammatory cytokines TNFα, IL-1β and NFκB in the lung tissue, as well as the development of pulmonary damages, while TLR4 knockout mice were resistant to PQ poisoning^36^. Consistently, we showed that in TLR4^−/−^ mice, not only the serum levels of IL-23 and IL-17A were significantly reduced, but also the neutrophil infiltration into the lung tissue was attenuated dramatically. Although we did not present direct evidence that PQ-induced HMGB1 directly acts on TLR4, our data, combined with previous findings, strongly support the involvement of TLR4 in HMGB1-induced IL-23 production and the subsequent lung injury. In addition to lung injury, the HMGB1-TLR4-IL-23-IL-17A axis has been reported to contribute to multiple disease models, including the cardiac ischemia-perfusion injury^17^, brain ischemia-perfusion injury^37^, drug-induced lethal hepatitis^11^. Given that TLR4 could mediate the signaling from multiple extracellular ligands and transduce the signals to multiple downstream targets, in addition to IL-23 and IL-17A, targeting TLR4 may provide broader therapeutic benefits, although may also be associated with more severe side effects, than targeting HMGB1, IL-23, or IL-17A alone. Furthermore, the significance of other TLRs, such as TLR2, in PQ poisoning is not explored in this study and thus should be addressed in the near future.

The importance of IL-23-IL-17A axis is well recognized in the pathogenesis of several autoimmune diseases, including inflammatory arthritis, psoriasis, and Crohn’s disease, and therefore the axis becomes an attractive therapeutic target in many ongoing clinical trials^38^. In this proof-of-principle study, we presented experimental evidence that targeting any component in the HMGB1-TLR4-IL-23-IL-17A axis is sufficient to alleviate lung injuries following PQ challenge, justifying its potential as the therapeutic target for correcting PQ-induced ALI.

The γδ T cells are a component of innate immunity and a significant producer of IL-17A in response to various cellular stress^34, 35, 39^. During the infections with many bacteria, such as *Mycobacterium tuberculosis*, *Escherichia coli*, and *Mycobacterium bovis*, γδ T cells may become the dominant IL-17A-producing cells, over Th17 cells^40–42^. In autoimmune diseases such as collagen-induced arthritis, a γδ T subset, Vγ4/Vδ4^+^ cells also potently produce IL-17A, exacerbating the disease^43^. In this study, we showed that the percentage of IL-17A-producing γδ T cells significantly increased in the lung tissue after PQ challenge, concomitant with PQ-induced ALI development. Depleting these cells significantly reduced yet not completely abolished IL-17A production, suggesting that although γδ T cells are an important source for IL-17A production in response to PQ challenge, other IL-17A-producing cells, such as Th17 cells, may also contribute to IL-17A production and ALI development in PQ poisoning.

In summary, this study demonstrates the significance of the HMGB1-TLR4-IL-23-IL-17A axis in neutrophil infiltration and ALI development following PQ challenge. It also provides evidence supporting the translational potential of targeting this axis in the treatment of PQ poisoning. However, this study mainly focused on one PQ dose, 40 mg/kg body weight, and on time point, 72 h after PQ challenge. Therefore, we have limited information on the dose response as well as temporal response of the HMGB1-TLR4-IL-23-IL-17A axis during ALI development following PQ challenge. Meanwhile, we realized that by targeting any component in the HMGB1-TLR4-IL-23-IL-17A axis, we achieved a significant yet not complete recovery of ALI-related symptoms, suggesting the existence of other parallel pathogenic mechanisms to PQ-induced ALI. Therefore, further studies should be performed to characterize the changes of the HMGB1-TLR4-IL-23-IL-17A axis during disease progression after different doses of PQ challenge and explore other molecular mechanisms associated with PQ-induced damages in the lung as well as other organs.

## Electronic supplementary material

Supplementary information
