# Supplementary material for: HMGB1-TLR4-IL23-IL17A axis promotes paraquat-induced acute lung injury by mediating neutrophil infiltration in mice
Source: Sci Rep. 2017 Apr 4;7:597. doi: 10.1038/s41598-017-00721-8 (PMC5429653; doi:10.1038/s41598-017-00721-8)
Supplement: Supplementary file 1 — Supplementary information [file 41598_2017_721_MOESM1_ESM.pdf]

Supplementary information

HMGB1-TLR4-IL23-IL17A axis promotes paraquat-induced acute lung injury by mediating neutrophil infiltration in mice

Bailing Yan[1], Feng Chen[2], Lijun Xu[3*], Jihong Xing[1] and Xuefu Wang[4*],

[1]Emergency Department, the First Hospital of Jilin University, Changchun 130021, China.

[2]Dermatology Department, China-Japan Union Hospital of Jilin University, Changchun 130033, China

[3]Department of Respiratory Medicine, the First Hospital, Jilin University, Changchun, 130021, China

[4]School of Pharmacy, Anhui Medical University, Hefei, Anhui 230032, China; Institute of Immunology, School of Life Sciences, University of Science and Technology of China, Hefei, Anhui, 230027, China.

*Corresponding authors:

Lijun Xu: Tel: +86 431 84808365. E-mail addresses: lijunxu001@sina.com.

Xuefu Wang: Tel: +86 13721051707. E-mail addresses: wangxuefu@ustc.edu.cn

Supplementary methods

Immunohistochemically test

The lung tissues were extracted from experiment and entrapped with paraffin. The test was made on the expression of the proteins of MPO in strict accordance with the operating instructions inside the kit of the reagent prepared. It was found through observation under microscope that yellow isotropic particles appearing in plasma of cell was positive. The kit was purchased from multiscience (shanghai, China).

Supplementary Figures and Legends

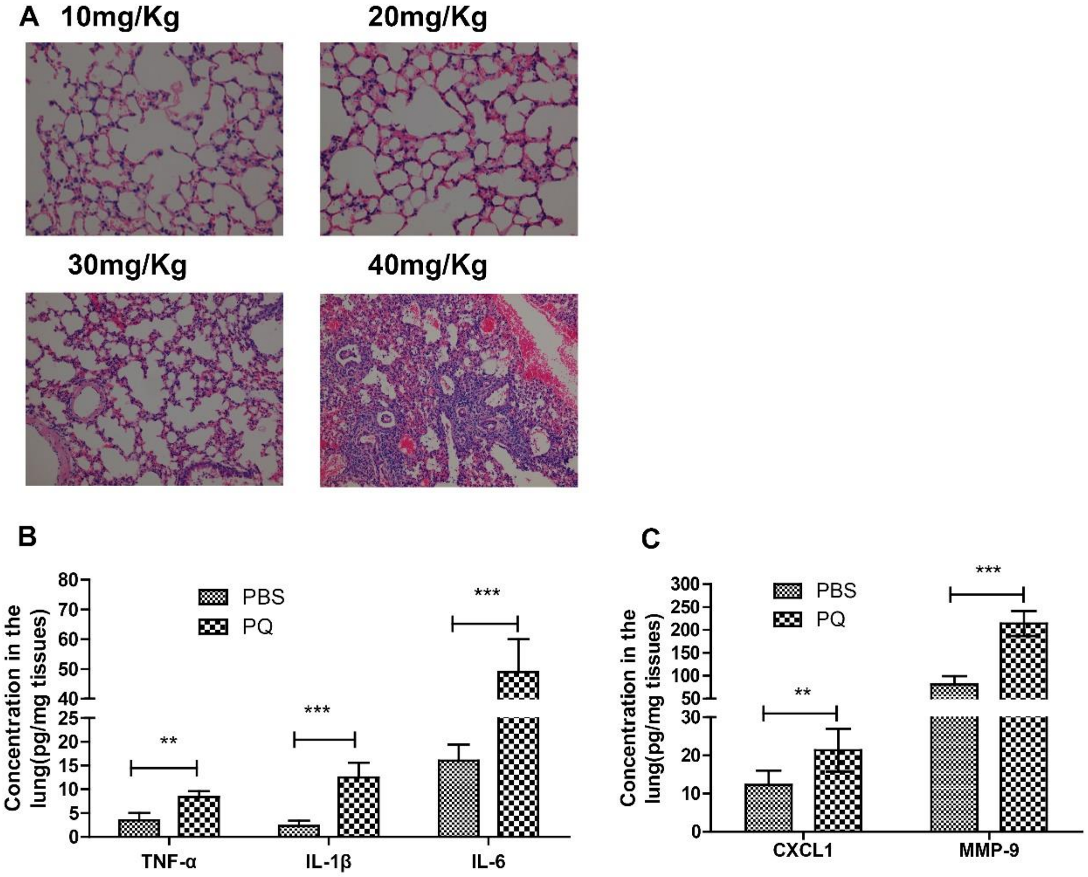

Fig. S1. PQ induces ALI and inflammatory response.

(A) Histological analysis of the mouse simulated with paraquat at dose of 10, 20, 30, 40mg/kg. The results show that PQ at the dose of 40mg/kg could make good model of acute lung injury. (B-C) The levels of TNF-a, IL-1β, IL-6, CXCL1, and MMP-9 production in the bronchoalveolar lavage fluid (BALF) was significantly elevated during paraquat (PQ)-induced acute lung injury (ALI), At 72 h after PQ or PBS challenge, the levels of TNF-a, IL-1β, IL-6 (B), CXCL1, and MMP-9 (C) in the BALF was measured by ELISA and compared between the two groups. Data were presented as mean ± SD. **$P < 0.01$; ***$P < 0.001$.

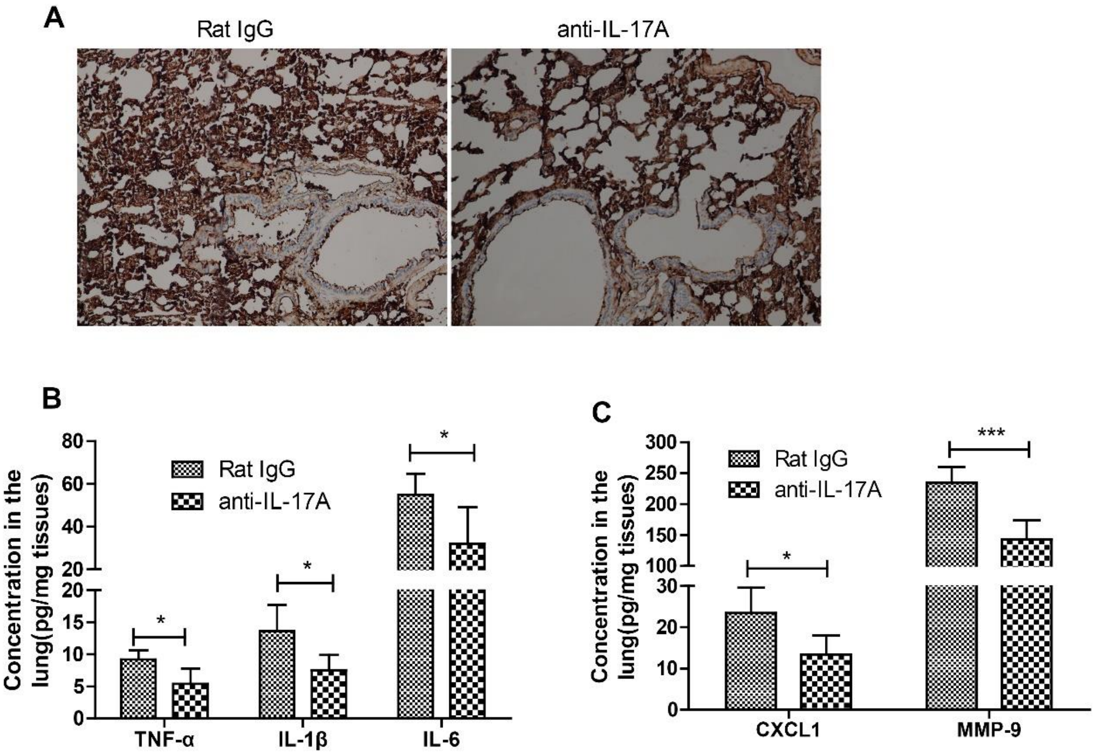

Fig.S2. Blocking IL-17A attenuates PQ-induced inflammatory response in lung.

(A) Immunohistochemical staining for MPO (200 ×). (B-C) Administration of anti-IL-17A decreased the levels of TNF-a, IL-1β, IL-6 (B), CXCL1, and MMP-9 (C) in BALF. Data are presented as means ± SD. *P < 0.05; ***P < 0.001.

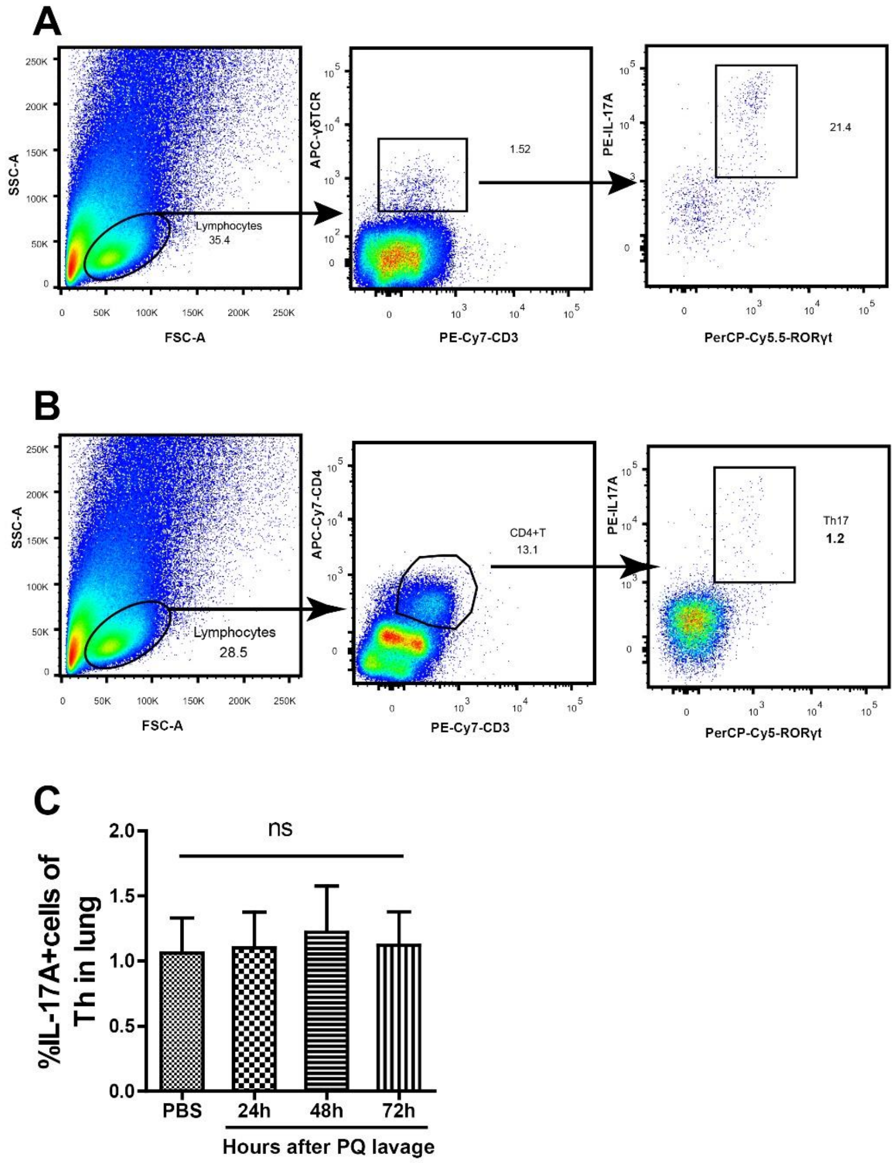

Fig.S3. The percentage of Th17 cells did not significantly change in PQ-induced ALI. (A) Gating strategy for the detection of IL-17A-producing γδT cells by flow cytometry. (B) Gating strategy for IL-17A producing Th17 cells. (C) The percentage of Th17 cells did not significantly change in the different time points. Data are present as means ± SD. ns means no significant difference.

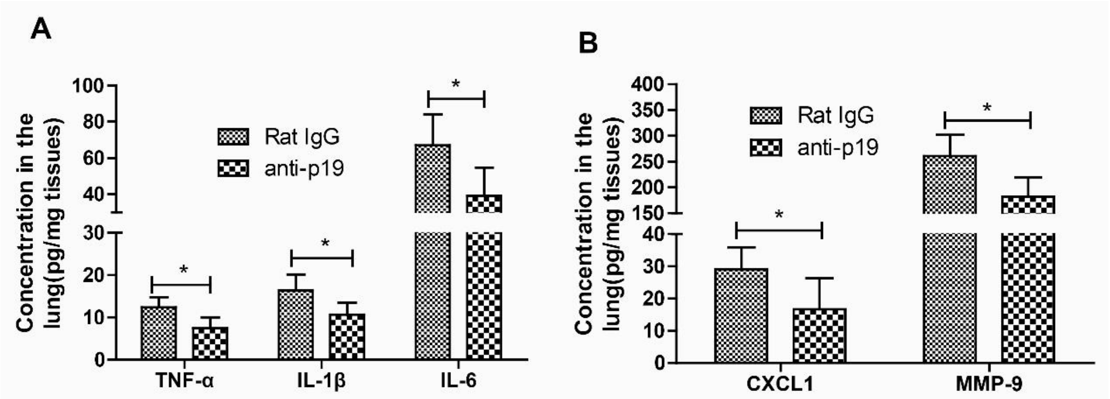

Fig.S4. Blocking IL-23 attenuates PQ-induced inflammatory response in lung.

Administration of anti-p19 significantly decreased the levels of TNF-a, IL-1β, IL-6 (A), CXCL1, and MMP-9 (B) in the BALF. Data are presented as mean ± SD. *P < 0.05.

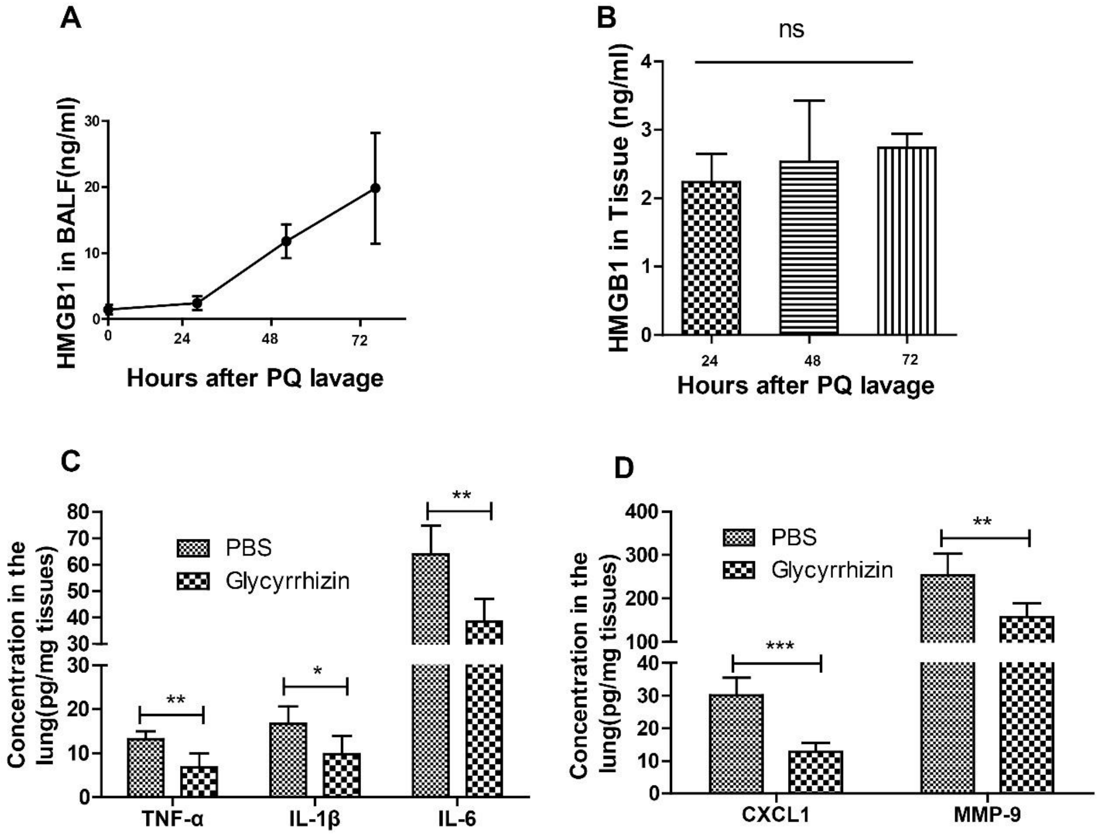

Fig. S5. Blocking HMGB1 attenuates PQ-induced inflammatory response in lung. (A-B) HMGB1 level in BALF (A) and gastric and intestinal tissues (B) at indicated time points after PQ lavage. (C-D) Pre-treatment of mice with Glycyrrhizin potently decreased the levels of TNF-a, IL-1β, IL-6 (C), CXCL1, and MMP-9 (D) in the BALF. Data are presented as mean ± SD. *$P < 0.05$; **$P < 0.01$; ***$P < 0.001$.
